# Supplementary figures and images for: The intermediate proteasome is constitutively expressed in pancreatic beta cells and upregulated by stimulatory, low concentrations of interleukin 1 β
Source: PLoS One. 2020 Feb 13;15(2):e0222432. doi: 10.1371/journal.pone.0222432 (PMC7018053; doi:10.1371/journal.pone.0222432)

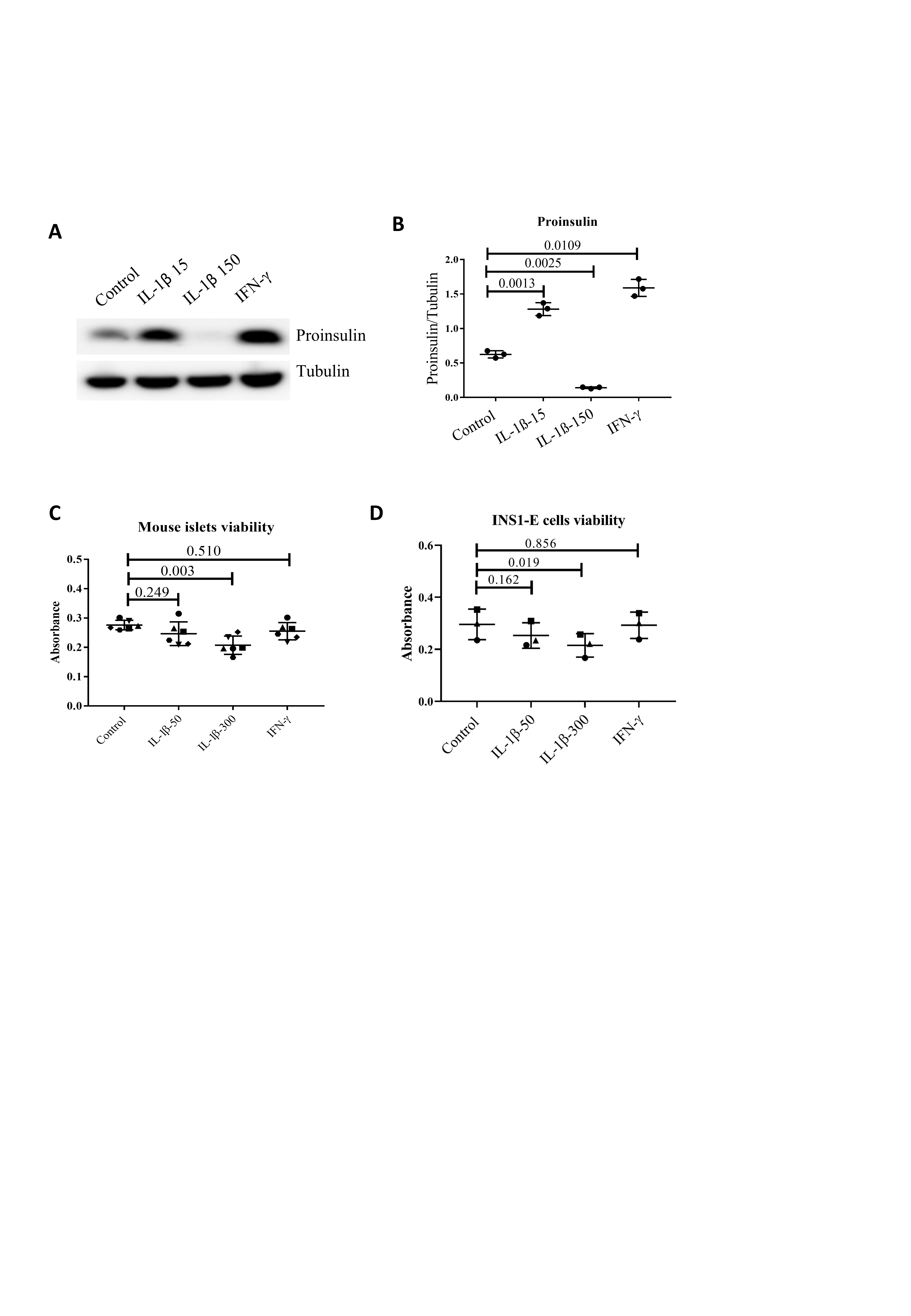

Supplement: S1 Fig — Lysates of cells exposed to IL-1β at low (15 pg/ml) and high concentration (150 pg/ml), IFN-γ (10ng/ml) and control medium, were run on SDS-PAGE and subjected to Western blotting in A) and proinsulin band intensity normalized to tubulin in B). C) and D) Mouse islets and INS1-E cell viability was tested. Staining reagent (AlamarBlue) was added to the cell culture for 4 h, incubated at 37oC, and the resulting fluorescence was read on a plate reader. Statistical analysis was performed by paired t-tests of treatments versus control. The data is shown as means with SD. (TIF) [file pone.0222432.s001.tif]

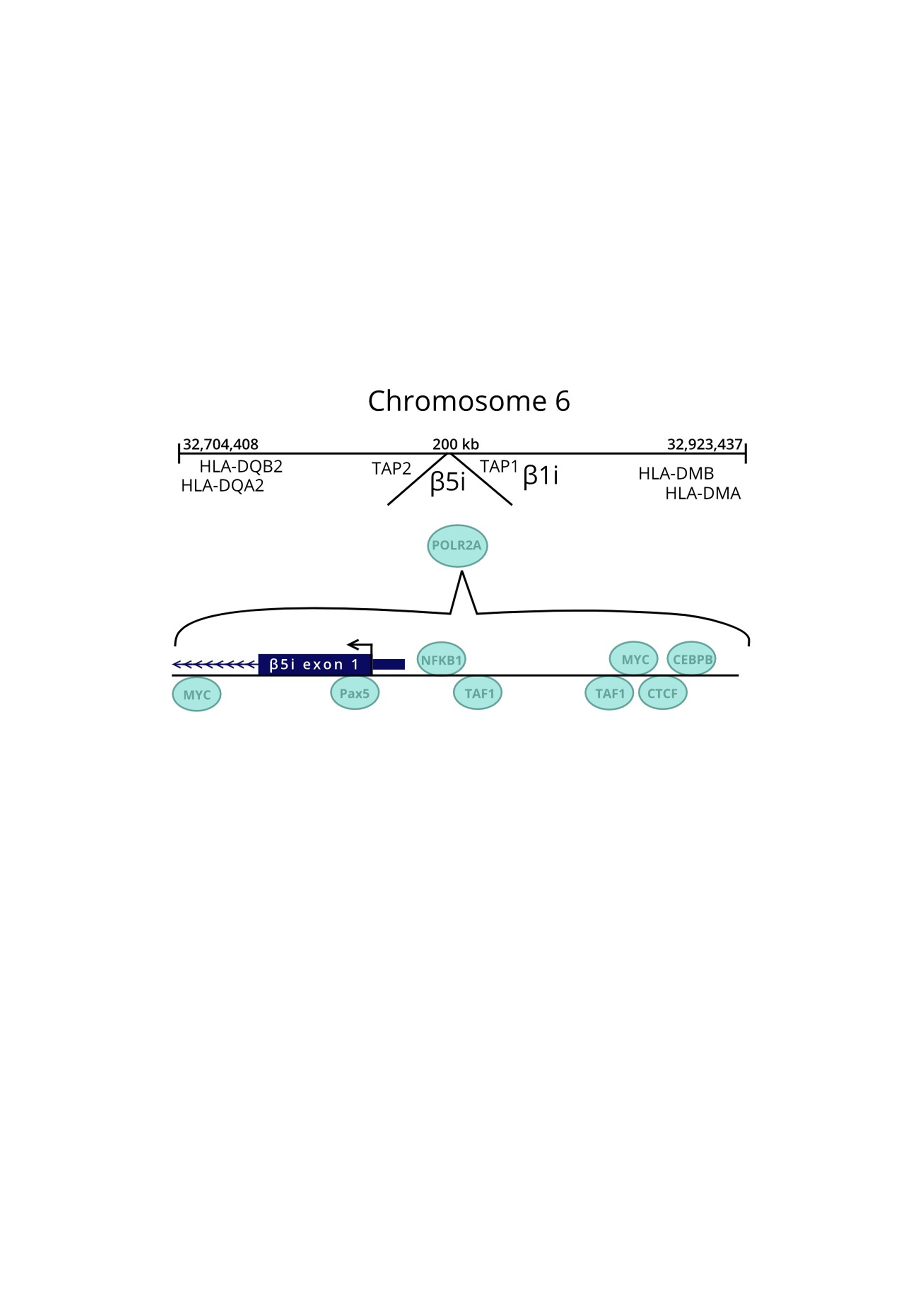

Supplement: S2 Fig — Genes for β5i (PSMB8) and β1i (PSMB9) reside in the MHC-II region on human chromosome 6. Presented transcription factors were included in UCSC genome browser genome GRCh37 and visualized with integrated regulation from ENCODE-track option. For clarity and in relevance to the current publication, only some transcription factors are presented. (TIF) [file pone.0222432.s002.tif]
