# Supplementary material for: The intermediate proteasome is constitutively expressed in pancreatic beta cells and upregulated by stimulatory, low concentrations of interleukin 1 β
Source: PLoS One. 2020 Feb 13;15(2):e0222432. doi: 10.1371/journal.pone.0222432 (PMC7018053; doi:10.1371/journal.pone.0222432)
Supplement: Attachment — Submitted filename: Response to Reviewers Khilji et al.docx [file pone.0222432.s003.docx]

**PONE-D-19-24067**

**The intermediate proteasome is constitutively expressed in pancreatic beta cells and upregulated by stimulatory, non-toxic concentrations of interleukin 1 b**

**Response to Reviewers:**

**Reviewer #1:** This is a descriptive study that examines the expression of subunits of the immune proteasome in pancreatic islets. The authors have used previously published RNAseq evidence to suggest that components of the immune proteasome are expressed at the mRNA level in b-cells and other islet endocrine cells. Overall the study is consistent with other reports showing immune proteasome expression under various conditions with new information suggestive that long exposures with low concentrations of IL-1 are capable of stimulating subunit expression.

There are a number of concerns that could be addressed or clarified in the text. Using RNAseq the authors provide in support of immune proteasome expressed in b-cells under basal conditions. How was RNAseq used to quantify mRNA levels?

*Two types of RNAseq data are included in the manuscript. Fig.1A presents human single cell sequencing data and Table 3 presents bulk mouse pancreatic islet RNA sequencing data.*

*Quantification of single-cell RNA sequencing of human pancreatic islets was performed through data analysis with bcbio-nextgen toolkit (https://bcbio-nextgen.readthedocs.io/en/latest/), followed by the Salmon algorithm (Patro, Duggal et al. 2017) for quantitation of mRNA counts. Data is then expressed as log2 of counts per million (CPM). The original use of data and more analysis details are included in, cited in the Material and Methods section, reference (Segerstolpe, Palasantza et al. 2016). Material and Method section has been amended to reflect changes.*

*Table 3 and its description (as well as manuscript text at the lane 203-216; all lane references refer to marked copy of the manuscript) has been modified to underlie the fact that data comes from bulk mouse islet sequencing (see below).*

Quantification of RNAseq? How is it possible to determine a p-value on these studies (Table 3) and how was quantification performed. Number of cells expressing the targets or the level of expression (i.e. number of reads?). Further, was this confirmed by PCR? Also, was this one isolation of islets and the n represent the number of cells sequenced, or were multiple isolations performed for each single cell sequence?

*We thank the Reviewer for raising this issue as it made us realize that the data should be reported in a different and more detailed way. Currently the data reports on in RPKM for the specific genes of 3 independent experiments as well as their means and is analyzed by Student’s paired t-test. The conclusions remain the same. As mentioned in the material and methods section Table 3 reports on whole islets bulk RNAseq and was obtained from 3 separate isolations of islets from several mice per condition. The method of quantification is described in detail and a reference to the original publication is provided. The expression of six genes reported in this study have not been validated by PCR but multiple other genes in the original study were for ex. insulin, Mafa, Pdx-1 and Ucn3.*

*Text changes can be found in lanes 203-216, 354-355 and overhauled Table 3 at lane 346*.

There is also some concern with the MS analysis of cross-linked proteasomes via immunoprecipitation approaches. It is not clear how the crosslinking on intact cells crosslinks the proteasome found in the cytoplasm (Fig 2). Some controls could be helpful with this analysis to support the authors conclusions.

*Co-author Marie-Pierre Bousquet’s group has set the original protocol to in vivo crosslink proteins on intact cells of various origins and then to very efficiently immmunopurify proteasome complexes after cell lysis. They have shown that this crosslinking strategy helps stabilizing Proteasome Interacting Proteins (PIPs), in particular important proteasome regulators, like the 19S and PA28 activators, and enables to characterize the whole diversity of proteasome complexes. In their 1st publication (Bousquet-Dubouch MP et al., Mol. Cell. Proteomics, 2009), the comparison of crosslinked cells and non-crosslinked cells (used as a control) has clearly demonstrated the benefit of formaldehyde crosslinking step on the efficacy of recovery of proteasome subunits and PIPs. Since then, the protocol has been successfully applied to study proteasome complexes in different subcellular compartments in leukemic cells (Fabre B. et al, Mol. Cell. Proteomics, 2013) and in cells and tissues of various origins (Fabre B. et al, J. Proteome Res., 2014; Fabre B. et al, Mol. Sys. Biol., 2015).*

*Material and method section has been amended to include the original publication for the protocol reference (lane 234).*

There is also concern regarding “non-toxic” concentrations of IL-1. It is not clear that mouse or human islets are sensitive to IL-1 alone and therefor all concentrations of IL-1 are non-toxic. It is suggested that the text be modified to low and high concentrations to be more consistent with the biological action.

*We agree with the reviewer and have corrected the text accordingly.*

Also- the merits of a 10 day exposure are not clear- what is the time- and concentration-dependence of IL-1 on immune proteasome subunit expression?

*We thank the Reviewer for raising this issue. The rational for doing the 10 days exposure to low doses of IL-1 was to mimic and better resemble the low-grade inflammation observed in diabetes. Although, even 24h exposure to IL-1 induces significant changes in proteasome subunit expression and activity the longer exposure, more characteristic for diabetes pathology could result in a loss of inducible subunits expression and induction of other detrimental to beta cells processes. However as reported in our collaborators publication (Ibarra et al. Mol Cell Endocrinol. 2019) prolonged exposure of mouse islets to IL-1 resulted in decrease of glucose stimulated insulin secretion but did not reduce cellular insulin content, did not induce endoplasmic reticulum stress or cell death and inducible subunits expression was maintained. This experiment provides a time perspective underlying the relevance of the observed short- and long-term subunits induction to the pathology of diabetes. We have modified the manuscript text at lane 502-511.*

**Reviewer #2:** The authors describe the constitutive expression of intermediate proteasomes in pancreatic beta cells. This expression is upregulated by interleukin 1b (IL1b). Mining of available RNA-seq data of single cells from pancreatic islets of healthy individuals and analysis of human and mouse islets, INS-1E (insulinoma), A20 (lymphoma) and Jurkat (leukemic cells) cells, revealed high expression of the inducible proteasome subunits in immune cells and low but consistent expression in the pancreatic cells. By LC-MS/MS analysis, proteasomes from INS-1E cells were classified either as standard (s-proteasomes) or beta5i-containing (14%) but no proteasomes were detected with the beta1i and beta2i subunits. With the exception of the A20 cells, all other cells types tested had proteasomes exhibiting mainly chymotrypsin-like activity (50-60%) followed by trypsin- and caspase-like activity. Incubation of human/mouse islets with ONX-0914 – an inhibitor of beta5i – led to reduction in chymotrypsin-like activity. Moreover, IL-1b upregulated the expression of the inducible subunits beta-1 (b1i), -2 (b2i) and -5 (b5i) and the proteasome activity in beta cells.

The findings are important as our knowledge of the proteasome activities in beta cells is still fairly limited. The authors provide a comprehensive picture of proteasome composition, especially with respect to the inducible subunits, in beta cells. Overall, the study is technically sound and the results support the conclusions. Statistical analysis of the data has been performed. I only have a few minor comments:

1. The authors should address the discrepancy in the level of chymotrypsin-like activity in the proteasomes of beta cells and Jurkat cells vs. A20 cells (lines 305-307). This is only mentioned in passing in the discussion (line 452).

*We have made changes to the manuscript to clearly underlie observed discrepancies and pointed to their possible sources although we have to admit that at this stage we do not understand the observed proteasome profile in A20 cells (lane 466-477). The proteasome activity and/or substrate specific activity can be regulated on multiple levels and the issue has been recently reviewed in Kors et al. Front. Mol. Biosci., 16 July 2019,* [*https://doi.org/10.3389/fmolb.2019.00048*](https://doi.org/10.3389/fmolb.2019.00048)*, although, again, it does not provide a clear explanation of our observation.*

2. Reporting the viability of cells (e.g. in figure 5) would be helpful to better assess the reported effects and the effect of treatment on the cells or islets.

*We agree with the Reviewer and have added data on mouse islets and INS1-E cell viability to the S1 figure and made appropriate changes in the manuscript text (lane 367-368) adding also references from previously published work e.g. Ibarra et al. Mol Cell Endocrinol. 2019 PMID: 31362031 Fig. 1E.*

***References***

Patro, R., G. Duggal, M. I. Love, R. A. Irizarry and C. Kingsford (2017). "Salmon provides fast and bias-aware quantification of transcript expression." Nat Methods **14**(4): 417-419.

Segerstolpe, A., A. Palasantza, P. Eliasson, E. M. Andersson, A. C. Andreasson, X. Sun, S. Picelli, A. Sabirsh, M. Clausen, M. K. Bjursell, D. M. Smith, M. Kasper, C. Ammala and R. Sandberg (2016). "Single-Cell Transcriptome Profiling of Human Pancreatic Islets in Health and Type 2 Diabetes." Cell Metab **24**(4): 593-607.
