# Supplementary material for: The intermediate proteasome is constitutively expressed in pancreatic beta cells and upregulated by stimulatory, low concentrations of interleukin 1 β
Source: PLoS One. 2020 Feb 13;15(2):e0222432. doi: 10.1371/journal.pone.0222432 (PMC7018053; doi:10.1371/journal.pone.0222432)
Supplement: Attachment — Submitted filename: Response to Reviewers Khilji et al Jan 21 2020.docx [file pone.0222432.s004.docx]

**PONE-D-19-24067**

**The intermediate proteasome is constitutively expressed in pancreatic beta cells and upregulated by stimulatory, non-toxic concentrations of interleukin 1 b**

**Response to Editor Comments**

The revised version of the manuscript addresses most of the comments raised by the original reviewers.

There are only two points that remained to be clarified in the manuscript:

• **The authors need to mention and justify that Normal distribution needs too be assumed for the use of parametric tests.**

The following text has been added to Statistical analysis section of the manuscript:

*All samples were selected without bias and represent biological not technical variations. Distribution of islets, specifically, were randomized and independent of e.g. size and shape. As a result, samples should be homogenous and represent biological variation, and both protein expression and activity is therefore assumed to be normally distributed (43, 44). Furthermore, normality of all expression data was tested with a Shapiro-Wilk test and found normally distributed and tested using a student t-test. Meanwhile proteasome activity and cell viability data is represented by a mean value of technical replicates, and as such should be normally distributed according to the central limits theorem (45).*

**• The authors need to emphasize and discuss in the text that the replicates are technical replicates (3 isolations) and not biological replicates.**

*The replicates presented in all figures, irrespective if those are cells or pancreatic islets, are biological* ***not*** *technical replicates.*

*Islet isolation: islets were isolated on separate days and from separate group of mice and that constitutes biological replications. Similarly, in case of human islets obtained from deceased donors, isolated islets represent biological variability (thus are replications) not a technical replicate.*

*Proteasome studies: for each data point cells were separately cultured (minimum two weeks), plated (on different days) and collected. That procedure constitutes biological replication for cell culture experiments. Then each data set was run, in minimum, as three technical replicates and the mean of those is presented.*

*We have amended the manuscript text to emphasize the fact that presented data is based on biological replicates. See lanes: 164-164, 287, 311 (already mentioned), 334-335, 361-362, 383, 403 (already mentioned).*
